# Association mapping by pooled sequencing identifies *TOLL 11* as a protective factor against *Plasmodium falciparum* in *Anopheles gambiae*

**DOI:** 10.1186/s12864-015-2009-z

**Published:** 2015-10-13

**Authors:** Seth N. Redmond, Karin Eiglmeier, Christian Mitri, Kyriacos Markianos, Wamdaogo M. Guelbeogo, Awa Gneme, Alison T. Isaacs, Boubacar Coulibaly, Emma Brito-Fravallo, Gareth Maslen, Daniel Mead, Oumou Niare, Sekou F. Traore, N’Fale Sagnon, Dominic Kwiatkowski, Michelle M. Riehle, Kenneth D. Vernick

**Affiliations:** Department of Parasites and Insect Vectors, Institut Pasteur, Unit of Insect Vector Genetics and Genomics, 28 rue du Docteur Roux, Paris, 75015 France; CNRS Unit of Hosts, Vectors and Pathogens, Paris, France (URA3012), 28 rue du Docteur Roux, Paris, 75015 France; Program in Genomics, Boston Children’s Hospital, Harvard Medical School, 3 Blackfan Street, Boston, MA 02115 USA; Centre National de Recherche et de Formation sur le Paludisme, 1487 Avenue de l’Oubritenga, 01 BP 2208 Ouagadougou, Burkina Faso; Malaria Research and Training Centre, Faculty of Medicine and Dentistry, University of Mali, Point G, Bamako, Mali; Wellcome Trust Sanger Institute, Hinxton, Cambridge UK; Wellcome Trust Centre for Human Genetics, Oxford, UK; Department of Microbiology, University of Minnesota, 1500 Gortner Avenue, Saint Paul, MN 55108 USA

**Keywords:** Mosquito, Malaria, Genetic analysis, GWAS, Host-pathogen interaction, Population genomics, Pooled sequencing

## Abstract

**Background:**

The genome-wide association study (GWAS) techniques that have been used for genetic mapping in other organisms have not been successfully applied to mosquitoes, which have genetic characteristics of high nucleotide diversity, low linkage disequilibrium, and complex population stratification that render population-based GWAS essentially unfeasible at realistic sample size and marker density.

**Methods:**

We designed a novel mapping strategy for the mosquito system that combines the power of linkage mapping with the resolution afforded by genetic association. We established founder colonies from West Africa, controlled for diversity, linkage disequilibrium and population stratification. Colonies were challenged by feeding on the infectious stage of the human malaria parasite, *Plasmodium falciparum*, mosquitoes were phenotyped for parasite load, and DNA pools for phenotypically similar mosquitoes were Illumina sequenced. Phenotype-genotype mapping was carried out in two stages, coarse and fine.

**Results:**

In the first mapping stage, pooled sequences were analysed genome-wide for intervals displaying relativereduction in diversity between phenotype pools, and candidate genomic loci were identified for influence upon parasite infection levels. In the second mapping stage, focused genotyping of SNPs from the first mapping stage was carried out in unpooled individual mosquitoes and replicates. The second stage confirmed significant SNPs in a locus encoding two Toll-family proteins. RNAi-mediated gene silencing and infection challenge revealed that *TOLL 11* protects mosquitoes against *P. falciparum* infection.

**Conclusions:**

We present an efficient and cost-effective method for genetic mapping using natural variation segregating in defined recent *Anopheles* founder colonies, and demonstrate its applicability for mapping in a complex non-model genome. This approach is a practical and preferred alternative to population-based GWAS for first-pass mapping of phenotypes in *Anopheles*. This design should facilitate mapping of other traits involved in physiology, epidemiology, and behaviour.

**Electronic supplementary material:**

The online version of this article (doi:10.1186/s12864-015-2009-z) contains supplementary material, which is available to authorized users.

## Background

The mosquito *Anopheles gambiae* is the principal vector of human malaria in sub-Saharan Africa, particularly of the most deadly malaria species, *Plasmodium falciparum*. Mosquito susceptibility to *P. falciparum* has a strong genetic component, indicated by population-based mapping of quantitative trait loci (QTLs) [1–3], and laboratory-based phenotypic selection of resistant lines [4]. However, the genome-wide association study (GWAS) techniques that have been used to map human [5, 6] and *Plasmodium* genes [7], have not been successfully applied to the mosquito.

Previous QTL mapping in wild *A. gambiae* pedigrees identified a cluster of loci that form the *Plasmodium resistance island* (PRI) [3], a region containing a number of novel immune genes. The PRI region was found to explain 89 % of the variation in resistance to *P. falciparum*. QTL mapping has high power to detect loci, but the extended linkage blocks reduce resolving power, and the 15 Mb PRI contains over 900 genes. Association mapping in a complex population in principle can resolve loci to many fewer genes. Population-based association testing of a small panel of candidate immune genes was applied to the mosquito in two studies [8, 9], which were limited because association requires large sample sizes, careful exclusion of population subdivision among the samples, and replication using independent phenotyped samples, which are all difficult in *A. gambiae.*

Successful GWAS is reliant on a combination of linkage, diversity, and allele penetrance. Linkage disequilibrium (LD) is a particularly important determinant of the power to detect a locus, while high levels of genetic diversity decrease the power of detection at a feasible sample size. Optimum statistical power is achieved when LD is highest (i.e., *r*^*2*^ = 1), the frequency difference between alleles is near 0, and the variation in phenotype can be explained by a single allele of strong effect. However, *A. gambiae* offers conditions far from optimal, with negligible levels of LD (*r*^*2*^ ~ 0.05 within 1 kb [10, 11]), and nucleotide diversity at least an order of magnitude greater than that found in human [12].

Modified experimental approaches combining the strengths of linkage mapping with the resolution of association mapping have been used in some model systems. Controlled-diversity colonies enable fine-resolution mapping under tractable genetic conditions in, for example, the mouse Diversity Outbred lines and the *Drosophila* Synthetic Population Resource [13–16]. Both of these approaches use material derived from advanced inter-crosses of inbred lines (often with known phenotypic traits), with breeding controlled to prevent excessive genetic drift. The challenge in *Anopheles* is similar, yet the context very different. In model organisms, the interest is to map a wide range of phenotypes within a series of highly controlled lines that still display sufficient phenotypic diversity. In comparison, vector biologists are more interested in particular phenotypes related to disease transmission such as pathogen interaction, behaviour and insecticide resistance, where it is desirable to capture natural variation. As well as clarifying the basic biology of mosquito-pathogen interactions and immunity, sampling natural alleles identifies natural factors that influence the epidemiology of malaria transmission. Any method used for diversity-controlled GWAS mapping should, therefore, provide a valid model for natural population structure of the species. That is, it should also represent a consistent panmictic subset of that population structure, as well as providing the same advantages as other controlled-diversity mapping approaches.

To this end, we developed a set of diversity-controlled *A. gambiae* founder colonies for use in genetic mapping and functional studies. The founder colonies are more favourable than wild mosquitoes for mapping because they display higher levels of informative LD, which facilitates detection of associations, they carry lower levels of genetic variation, which also augments the power of detection, and population subdivision is controlled to avoid the risk of spurious associations due to stratification. Each founder colony was initiated with the eggs of ~10 wild-caught females from West Africa, which were genotyped for markers of population structure before combining into the defined founder colonies. Using the founder colonies, we carried out a GWAS study of *Anopheles* susceptibility to infection with *P. falciparum*. The first stage used Illumina sequence of infection phenotype pools as genotyping, in order to rapidly highlight candidate loci. In the second stage, mosquitoes were individually genotyped with a panel of informative SNPs filtered from the candidate loci, for technical validation, replication, and fine mapping of the association locus. Finally, candidate genes were identified and functionally tested. An important advantage of the founder colony strategy as compared to GWAS in wild populations is that functional follow-up studies can be done in the same genetic background before attempting to type candidate variants in the wild population.

## Results

### Initiation and genetic description of mapping populations from nature

We initiated founder colonies of *A. gambiae* M-form mosquitoes from the West African population (Burkina Faso and Mali) as a tool to reduce genetic complexity and enhance informativeness for mapping by pooled sequencing. The approach relies on unsupervised mating, each colony initiated with the progeny of ~10 wild-captured gravid female mosquitoes. The founder mosquitoes assortatively mated in nature, thus ensuring that the progeny reproduce the genetic subdivisions of the natural population.

**Fig. 1:**
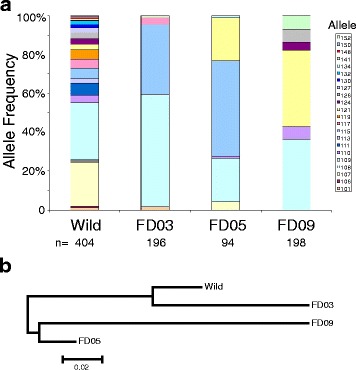
Genetic diversity of West African founder colonies. **a** Allele frequencies for microsatellite marker H603. The wild population segregates 22 alleles, while each founder colony segregates a distinct subset of six alleles. Only one wild allele is shared among all three founder colony. Sample sizes (n) are indicated as numbers of alleles tested. **b** Neighbor-joining trees based on pairwise Fst values indicate that the colonies have captured distinct subsets of variation from the source wild population. All branches are significantly greater than 0

The genetic composition of founder colonies was determined by microsatellite typing. First, we compared genotypes for a highly polymorphic microsatellite, H603, located at 42 Mb on the left of the chromosome 2. Each of three founder colonies tested segregate six marker alleles, as compared to 22 alleles at this locus in the wild source population (Fig. 1a). Overall, a total of 13 of the 22 wild alleles were detected among the three founder colonies. Thus, any individual founder colony maintains and segregates limited variation, but the sum of multiple such colonies may approximate and serve as a proxy for the genetic diversity of the natural population. Only one allele by state (no claim is made about descent), the 109 nt allele (turquoise in Fig. 1a), is shared by all three founder colonies, indicating that founder colonies capture largely distinct subsets of natural variation. Interestingly, alleles that are rare in nature not only persist but can achieve appreciable frequency within a founder colony. Thus, analysis of multiple founder colonies queries common and rare variants from the population.

Analysis of genetic variation and sharing between colonies using five microsatellite markers further indicates that the artificial population bottleneck creates the advantages of founder populations, which have been extensively discussed in human genetics [17, 18]. The colonies are distinct but related synthetic populations, with varying levels of Fst among colonies and the wild source population (Fig. 1b). Thus, the founder colonies perform as a complexity-reduction tool for tractable genetic query of both frequent and rare natural allele frequency classes under controlled laboratory conditions and at reasonable sample sizes, but query a greater amount of variation than single-pair crosses.

We derived an estimate of the genetic mapping resolution of the founder colonies. The crossovers from a given point (e.g., the trait locus) are exponentially distributed with rate Νρ, where Ν is the number of crossovers and ρ is the crossover rate [19], about one Mb per cM in *A. gambiae* [11, 20]. The expectation for the interval containing flanking crossovers around the trait locus is 1/Νρ from each side. Therefore, when 10 individuals share the same variant under a dominant model, and ignoring the few homozygotes, the maximum resolution will be 2/Νρ = 2/nkρ, where n is the number of mosquitoes, k is the number of generations since the founding event. The current founder colonies were propagated for at least 30 generations since initiation, thus the expected resolution for a fully informative phenotype is at least 2/30*10*1 = 2/300 = 0.7*10^−2^ = 0.7 cM. This is a genomic average estimate of resolution, which will vary empirically according to the recombinational properties of different regions of the genome. In addition, loss of information due to incomplete penetrance of a trait would also extend the resolved mapping interval.

### First-stage mapping by pooled sequencing identifies candidate loci

Mosquitoes from two founder colonies, Fd03 and Fd09, were challenged by feeding on cultured *P. falciparum* gametocytes, and individual mosquito infection phenotypes were determined by dissection and counts of midgut oocysts. DNA from mosquitoes with similar phenotypes were combined and Illumina-sequenced as pools. A quantitative description of the pools is given in the methods. Sequences of pools were compared across the genome to detect regions displaying reduction in haplotype diversity, in order to detect candidate intervals carrying variants that underlie the phenotype. By definition, these decreased heterozygosity candidate intervals will be enriched for haplotypes carrying the causative haplotype, and other non-causative haplotypes will be simultaneously depleted from the same phenotype pool. Regions of the genome not associated with the phenotype should display random segregation of haplotypes across pools.

**Fig. 2:**
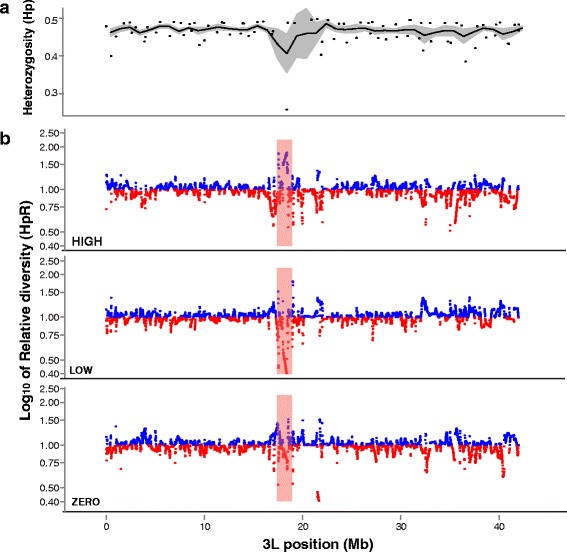
Genome-wide mapping of candidate genetic loci by measurement of relative heterozygosity in sequenced phenotype pools. Plots depict heterozygosity measures on chromosome 3 L. a Total pooled heterozygosity (*Hp*) was calculated in a sliding 10 kb window along chromosome 3 L within the Fd03 founder colony. Dots indicate minimum and maximum values for a 1 Mb window, the black line indicates the average heterozygosity and the gray shading represents the standard deviation of total pooled heterozygosity across a 1 Mb window. b Relative diversity (*HpR*) per 1 Mb window, calculated as the proportion of heterozygosity in a given pool relative to total heterozygosity within the source Fd03 founder colony. Color of point indicates per window elevated heterozygosity (blue lines), or reduced heterozygosity (red lines), plotted as log base 10 of the relative diversity. Phenotype pool identity is indicated in the lower left of each panel (high, low, zero oocysts). A relative heterozygosity value of *HpR* = 1 indicates the same heterozygosity levels in tested pool as compared to all other pools, values of *HpR* > 1 indicate greater heterozygosity in the tested pool and values of *HpR* < 1 indicate lower heterozygosity in the tested pool. On the log scale y-axis, values of 0.5 and 2.0 are equidistant from 1. Candidate locus 3.1 is indicated by the red vertical shaded bar at coordinates 17.4-19.1 Mb. In this interval, relative heterozygosity is increased in the high pool and simultaneously reduced in the low and zero pools

The first mapping stage was comprised of genome-wide ascertainment of candidate loci. Pooled heterozygosity (Hp) was calculated across sliding windows for each of the phenotype pools individually, as well as total heterozygosity for the whole founder colony combined. Relative diversity (*HpR*) was calculated as the proportion of heterozygosity in a phenotype pool relative to total heterozygosity within the whole founder colony after normalising for overall read-depth in each pool. Standard deviation of *HpR* values (*SHpR*) was used to identify regions with over-represented haplotypes in a given phenotype pool by comparison to the same positions in the whole founder colony. The measurement of heterozygosity within a pool was done in comparison to the same positions in the whole founder colony, and thus was normalized for local variation of heterozygosity across the genome. The analysis yielded three candidate loci in two different founder colonies, located at chromosome:coordinates, 3 L:17409-19071 kb (colony Fd03), 2R:17385-26524 kb (colony Fd09), 2R:47490-60531 kb (colony Fd09). These candidate loci were named 3.1, 9.1, and 9.2, respectively. Plots of candidate locus 3.1 are shown in Fig. 2 (chromosome 3 L) and Additional file 1: Figure S1 (chromosomes 2 and X). Plots of candidate loci 9.1 and 9.2 are shown in Additional file 2: Figure S2 (all chromosomes). Candidate locus 9.1 is coincident with the 2Rb paracentric chromosomal inversion.

In order to estimate significance values for mapped loci, a permutation analysis was carried out on the *SHpR* values by reselecting allele frequencies randomly from each of the phenotype pools. After 1000 tests, the selected *SHpR* regions were found to have median *SHpR* values that were within the 99.9th percentile of those selected randomly (equivalent to a *P*-value of 0.001).

### Functional description of candidate loci

Coding sequences within candidate loci were analysed for enrichment of Gene Ontology (GO) predicted functional categories. The two candidate loci from Fd09 contain 609 genes for candidate locus 9.1 and 708 genes for candidate locus 9.2. While the large number of genes in the two Fd09 loci might reduce the probability of detecting significant enrichments, both Fd09 loci demonstrated enrichment for genes with potential immune functions, with highly significant enrichment (*P* = 7.8e-6) for monoxygenase function in candidate locus 9.1, and the presence of multiple peroxidases in candidate locus 9.2, consistent with either a detoxification or ROS-based immune response. Analysis of genes with significantly enriched GO terms in candidate locus 9.1, however, indicated that most of these genes belong to a single cytochrome P450 cluster between 17.4 and 21.1 Mb.

Due to the coincidence of candidate locus 9.1 with the 2Rb chromosomal inversion, molecular karyotyping was carried out on all Fd09 samples. There was only one 2Rb/b homozygote in the high pool, with heterozygotes occurring randomly between all three pools (giving 2/3/3 copies of the inversion in zero/low/high pools respectively; each pool was comprised of 20 mosquitoes, thus 40 chromosomes). Thus, there was low power to test, but also no evident support for association of the frequency of 2Rb inversion genotypes or alleles with membership in phenotypic pools. For all mapped loci, we have tested for chromosomal inversions, and the *SHpR* method controls internally for sequence read depth by comparing each pool to the whole founder colony. Therefore, estimates of relative diversity should be robust to potentially confounding sources of local genome variation. The Fd03 candidate locus 3.1 contains only 74 genes, the majority of them with no functional information. Genetically-based candidate gene ascertainment would require deeper pooled sequence data, or ideally sequence variation data from phenotyped individuals, resources that have not been generated. Moreover, because the haplotype contains irrelevant as well as relevant SNPs in linkage, resolution by fine mapping may not be productive without propagation to generate additional recombinations. Consequently, we performed ad hoc ascertainment of candidates based on recognizable predicted gene function and other evidence. Of those with characterized function, two encode Toll-family proteins, *TOLL 10* and *TOLL 11* (AGAP001187, AGAP001186).

### Second-stage fine mapping of candidate loci

The second mapping stage comprised candidate locus confirmation and prioritization of candidate genes. SNPs within the candidate loci displaying the greatest difference in minor allele frequencies between any two phenotype pools were selected for genotyping in individual mosquitoes. SNPs were selected based on sequence from individual founder colonies, and were tested only within the same founder colony. A total of 44 SNPs were chosen from Fd09 across candidate loci 9.1 and 9.2, and 23 SNPs from Fd03 for candidate locus 3.1.

For each founder colony, fine-mapping was performed by genotyping all of the unpooled individual mosquitoes from the original infections from which the initial phenotype pools were constructed. For Fd03, an independent replicate infection, which did not contribute to the sequenced phenotype pools, was also genotyped. These two replicate infections were assessed by logistic regression separately, and where the odds ratio indicated the same effect, also together as one experiment (all Fd03 logistic regression values are given in Additional file 3). The individual typing of deconvoluted pools confirms that the pooled analysis results are recapitulated by individual genotyping, and serves as technical replication, while the typing of new material from the same colony but a completely independent infection serves as biological replication.

**Fig. 3:**
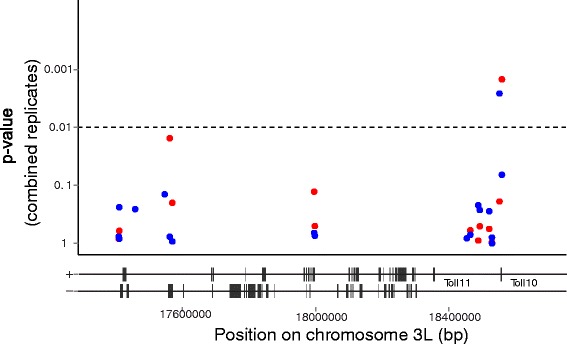
Individual genotyping confirms nucleotide variation significantly associated with P. falciparum infection outcome. Fine mapping by logistic regression analysis of Fd03 colony SNPs within locus 3.1 using individual mosquito SNP genotypes. Association is calculated across both biological replicates (the original infection used in pooled sequencing and genotyping of individuals deconvoluted from the pools, and a second independent infection of the same colony). Red points indicate p values for association with the phenotype, oocyst intensity (comparison between individuals from high and low pools), blue points indicate p values for association with oocyst infection prevalence (comparison between individuals from zero and all infected (combined low + high) pools). The dashed line indicates the significance threshold (*p* = 0.01). Genes within the locus are indicated beneath the plot; genes on the forward (+) and reverse (−) strands are shown separately, positions of *TOLL 11* and *TOLL 10* genes are indicated

Candidate locus 3.1 contained two SNPs with significant association after permutation, influencing both oocyst infection prevalence and intensity (Fig. 3, Additional file 4). The interval of candidate locus 3.1 was thus considered confirmed as a *P. falciparum* control locus, and following convention [3] is named Pfin7 (*Plasmodium falciparum* infection locus 7). The variant at 3 L:18559884 is a C:A mutation in the intergenic region between *TOLL 11* (AGAP011186) and *TOLL 10* (AGAP011187) with an odds ratio of 7.79 for oocyst infection intensity (*p* = 0.00148, calculated across both replicates). The variant at 3 L:18552220 is a T:C mutation located immediately downstream of *TOLL 10*, with an odds ratio of 3.15 for oocyst infection prevalence (*p* = 0.002594, calculated across both replicates). Individual genotyping did not confirm association with infection phenotype for either of the chromosome 2R candidate loci, 9.1 and 9.2, and they were not analysed further. The size of the locus 3.1 interval, ~2 Mb, is broadly consistent with the above theoretical prediction of genetic resolution in the founder colonies.

### *TOLL 11* displays protective function against *P. falciparum*

**Fig. 4:**
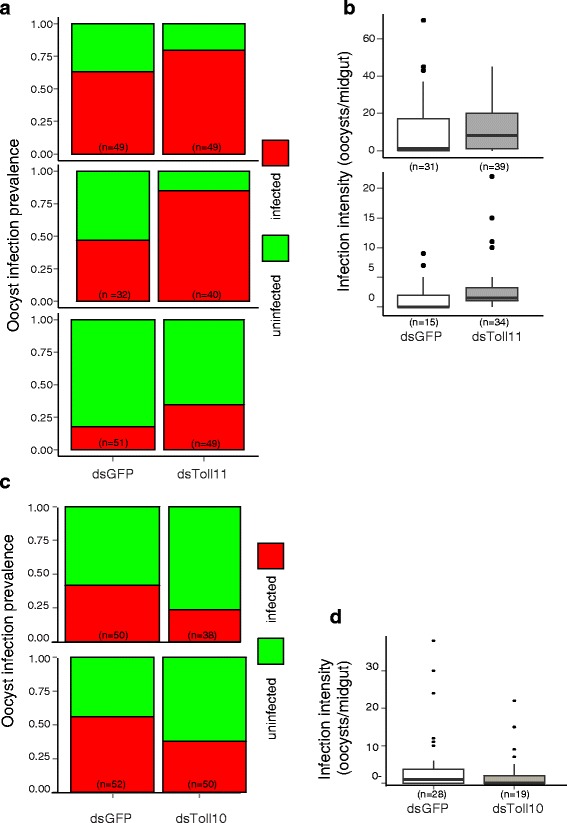
*TOLL 11* activity protects mosquitoes against infection with *P. falciparum*. Gene silencing of TOLL 11 followed by parasite challenge causes increased *P. falciparum* oocyst infection prevalence in *A. gambiae*. Silencing of TOLL 10 does not significantly alter infection phenotypes. **a** Prevalence of oocyst infection for Fd03 colony individuals treated with control dsRNA (dsGFP) or with dsRNA directed against TOLL 11 (dsTOLL11) and challenged with *P. falciparum* in 3 replicate experiments (replicate number in grey box). Green bars, proportion of uninfected mosquitoes (0 midgut oocysts), red bars, proportion of mosquitoes with ≥ 1 midgut oocyst. *P*-values are calculated using a *χ* ^2^ test across treatments. **b** Intensity of infection measured by the number of oocysts in midguts after dsRNA treatment and infection challenge. Intensity is only analyzed in mosquitoes with ≥1 midgut oocyst. Significance is calculated by the non-parametric Wilcoxon rank-sum test. For both infection prevalence and intensity, *P*-values across replicates for intensity were combined by the method of Fisher (see Methods). (**c**) and (**d**) as in (**a**) and (**b**), but with dsRNA directed against TOLL 10 (dsTOLL10). The number of replicates shown for oocyst intensity is less than for oocyst prevalence, because oocyst intensities were only statistically compared when the infection prevalences were above 30 % for both control and target gene silencing conditions. This cut-off provides for adequate statistical power for detecting differences in infection intensity

Bioinformatic filtering based on interpretable predicted gene functional and other evidence of the 73 predicted coding sequences within Pfin7 (Additional file 5: Table S3) prioritized two genes encoding Toll-family proteins, *TOLL 10* and *TOLL 11*. A functional test of *TOLL 11* effect by RNAi-mediated gene silencing followed by challenge with *P. falciparum* reveals that *TOLL 11* mediates significant protection against oocyst infection (Fig. 4). Silencing of *TOLL 11* caused an increase in oocyst prevalence of 16-38 % across three replicates (mean 24.5 %). The consistent increase in infection prevalence across three replicates was highly significant (*p* = 0.0008, p-values combined by the method of Fisher; individual replicates *p* = 0.118, 0.001, 0.086 Fig. 4a). Loss of *TOLL 11* function incurred a mean risk ratio for oocyst infection of 1.71 (comparison of infected and uninfected categories; individual replicates = 1.26, 1.81, 2.08 respectively). There was no effect of *TOLL 11* silencing upon a distinct phenotype, oocyst intensity (*p*-values = 0.850, 0.848; combined = 0.957, Fig. 4b).

*TOLL 10* did not show a significant effect for either infection prevalence or intensity (Fig. 4c, d). Uninfected/infected risk ratios (0.56, 0.69, mean = 0.63) did not indicate a phenotype for infection prevalence, (*p* = 0.117, 0.188, combined = 0.106, Fig. 4c). Results for infection intensity were also negative (*p* = 0.538, Fig. 4d). Although not significant, the results displayed a tendency that could be suggestive of a weak phenotype in which reduction of TOLL 10 transcript levels may lead to a lower infection prevalence, opposite to the TOLL 11 phenotype. Further work would be necessary to determine whether *TOLL 10* may display a significant phenotype under other conditions or genetic backgrounds.

Toll-family proteins are defined based on shared structural features [21]. Despite structural relatedness with the Toll receptor, TOLL 1, the other Toll-family members have not been well-characterized, and are not necessarily immune receptors. Even in Drosophila, functions of Toll-family proteins or their signaling pathways, besides TOLL 1, are also largely unknown [22]. In Anopheles, TOLL 1 and the Toll pathway are required for protection against rodent malarias, *P. berghei* and *P. yoelii*, while protection against the human malaria parasite *P. falciparum* is dominated by the IMD pathway [23, 24]. To our knowledge, the current results are the first report of a Toll-family member displaying protective function against *P. falciparum*. This also represents one of the rare reports of immune function for Toll-family proteins other than TOLL 1. TOLL 11 remains a candidate gene for control of *Plasmodium* susceptibility, and future work will be necessary to determine whether naturally occurring genetic variants in TOLL 11 are associated with differential susceptibility to *P. falciparum*.

## Discussion

We present a novel method of mosquito genomic mapping by pooled sequencing using recently initiated *A. gambiae* founder colonies, and demonstrate its applicability for mapping in a complex non-model genome. Genome-wide mapping identified three candidate loci, and fine-mapping by individual genotyping confirmed one of these, named Pfin7, which was significantly associated with control of oocyst infection prevalence. Functional testing of candidate genes encoding two Toll-family proteins, *TOLL 10* and *TOLL 11*, revealed a significant influence of *TOLL 11* upon *P. falciparum* oocyst infection prevalence.

### Mapping design

The founder colony mapping design allows us to sample natural variation with minimal breeding effort, and facilitates follow-up experiments under defined laboratory conditions. The limited diversity resulting from the founding event leads to simplified genetics, a limited number of founder alleles, and long haplotypes as compared to an association study based on field populations. Distinct from the “laboratory colonies” commonly used in mosquito genetic studies, however, some of which are decades old, with unknown histories of contamination and admixture, here we initiate multiple defined independent recent colonies from a local population that segregate stable inventories of alleles and consistent ancestral haplotype blocks per colony. Overall the design aims to balance advantages and limitations between two extremes in genetic mapping: i) GWAS in field populations, and ii) linkage mapping with isogenic lines.

The presented mapping design has a number of significant advantages over previous approaches and provides a basis for future association studies in mosquitoes. As compared to association mapping at the level of individual mosquitoes, our method using founder colonies and pooled sequencing requires only moderate expenditure for both sequencing and genotyping, although the benefits are not limited just to cost savings. *A. gambiae* is a technically difficult subject for genomic association mapping. Low levels of LD impair the power of locus detection, while high levels of diversity and the number of statistical tests necessary to query all diversity weaken the ability to detect a significant signal. The mosquito founder colony design used in this study alleviate both of these problems. The founder effect generated by initiating colonies from limited numbers of wild chromosomes increases informative LD, which augments power of locus detection, while simultaneously reducing the level of genetic diversity to a tractable level. At the time of colony initiation, the minor allele and minor haplotype frequencies were ~0.05, indicating that the founder bottleneck effectively created a mapping population with a large number of SNPs that perfectly type their haplotypes. The continued propagation of these strata-controlled colonies will aid subsequent follow-up studies, because functional assessment of candidate genes can be carried out in the same genetic background.

Pooled sequencing is in a cost trade-off with individual sequencing, which would be maximally informative but is currently cost-prohibitive for routine use. The main weakness of pooled sequencing is that it is difficult to measure allele frequencies, which can decrease efficiency of ascertainment of informative SNPs for fine mapping. However, the practical consequence is not severe, because a reduced conversion rate from predicted assay to typable SNP is overcome merely by testing more SNPs. An area of improvement in the future could be to analyze enrichment and depletion of particular alleles from extreme phenotype pools, which would not require exact measurements of allele frequencies within pools, but rather a comparison of allele frequencies across pools.

### *TOLL 11* as host-protective factor

Genetic mapping identified a small locus containing two Toll-family proteins, neither of which have a previously described phenotype. Functional tests revealed a protective phenotype against *P. falciparum* oocyst infection prevalence for *TOLL 11*. The Toll family possess a single transmembrane domain, a leucine-rich repeat region of variable size, highly divergent extracellular N-terminal and a conserved C-terminal intracellular TOLL-ILK1 domain [21]. The *TOLL 10* and *TOLL 11* genes identified in this locus together represent a species-specific expansion in *A. gambiae*, without orthologs in *Drosophila*.

The canonical Toll-family member, *TOLL 1*, has been extensively characterized in *Drosophila*. In addition to a role in embryonic pattern formation, the Toll pathway is activated in response to pathogens, including fungi and gram positive bacteria [22]. Only a few other Toll family members have described phenotypes, including anti-fungal and anti-viral functions for *Drosophila TOLL 5* [25] and *TOLL 7* [26], respectively. Studies in *Aedes aegypti* also indicate an antiviral role for the Toll pathway [27]. The precise mechanism of TOLL11 influence upon Plasmodium infection will require detailed functional studies. As with any immune factor, TOLL11 may bind in a complex with other immune factors, cross-talk with other signaling pathways, change the midgut microbial flora and/or alter mosquito behavior, to name several possible considerations. In any case, the decrease in TOLL11 transcript by gene silencing influences development of P. falciparum in the mosquito.

Two of the 11 *Anopheles* Toll family genes are thought to play immune roles, with *TOLL 1* and *TOLL 5* activity against gram positive bacteria [21], and an antifungal role for *TOLL 5* [28]. *TOLL 10* and *TOLL 11* display differential transcriptional regulation during embryonic development [29] and both genes are upregulated in response to bloodfeeding [30]. *TOLL 11* (though not *TOLL 10*) has further been shown to be differentially regulated in response to infection with *P. berghei* and *falciparum* [31]. The expression profile suggested a potential anti-Plasmodium role for this locus, but two previous attempts to associate variants in *TOLL 10* and *TOLL 11* with oocyst number generated negative results [8, 9]. The success of any association study depends on the selection of SNP markers with sufficient genetic informativeness, as discussed above. In the absence of comprehensive haplotype maps from which informative tagging SNPs could be derived, the greatest success will be achieved by a method that employs artificial haplotype creation or control and that genotypes with SNPs from the same population, as we have done here.

Follow-up studies of the Pfin7 locus in the natural population would be necessary to query the phenotypic penetrance of the locus, including sensitivity to epistasis with host genetic background and parasite genetic variation. Another reason to study the population is to determine the natural allele frequency of the locus. For that purpose, a high-density marker map of the Pfin7 locus could be used to type wild collections, in order to identify a core haplotype and measure its frequency.

## Conclusions

*Anopheles* genetic association and mapping studies in nature remain a challenge. Wild samples must be carefully typed for population structure and likely other confounding factors such as polymorphic inversions, which means that a large excess of samples need to be collected in order to assemble sufficient numbers of the targeted panmictic group. Another challenge is phenotyping of the wild samples. For malaria infection, wild-infected samples could be used, but this introduces variables such as mosquito age, host preference, and survival that, at best, demand larger sample size to overcome the noise, and at worst could introduce spurious results. Alternatively, wild samples can be phenotyped by experimental infection with blood from gametocyte carriers as we have done previously [3, 32], but this is technically demanding, and constitutes research with human subjects. Consequently, we explored the potential of colony-based representation of wild populations. We propose that controlled-diversity mapping populations, such as the founder colony design used here, are the preferred means for the foreseeable future for first-pass mapping of novel genomic regions of importance for *Plasmodium* resistance and other phenotypes in *Anopheles*, with possible follow-up in the natural population as appropriate.

## Methods

### Mosquito founder colonies

Wild caught *A. gambiae s.l.* females originated either from Burkina Faso (Goundry region) or from Mali (Bancoumana region). Gravid females were captured by aspiration indoors, ensuring that at the time of capture they had already mated assortatively under natural conditions and bloodfed. They were then placed individually in oviposition tubes with wet filter paper. Females that laid eggs were collected and stored in ethanol before genomic DNA extraction. Eggs from individual oviposition were placed in a pan of water with Tetramin fish food. Emerged adults were reared under standard conditions at 26 °C and 80 % humidity, 12 h light/dark cycle with access to cotton soaked in 10 % sucrose solution.

Females that laid eggs were typed for species, molecular form and the molecular karyotype of the 2La chromosomal inversion [16]. Maternal genotype was determined by genotyping. Because mating occurred in nature, the paternal genotype was inferred by genotyping F1 offspring. Isofemale families identified as *A. coluzzii* (M form) with the karyotype 2La/a, were used to initiate colonies. No hybrid families resulting from MxS form crosses were seen. Founder colony 03 (Fd03) was started with the F1 offspring from six mated females originating from Mali and founder colonies 5 (Fd05) and 9 (Fd09) were created with the offspring of 10 and 11, respectively, females from Burkina Faso. Colonies are routinely monitored for species and 2La inversion karyotype.

Individual mosquitoes from founder colonies Fd03, Fd05, and Fd09 were genotyped for five microsatellite markers (2 L.17686896, 2 L.19444747, 2 L.41431233, 2 L.40133863, and H603) using described primers and methods [3]. The naming convention of the indicated microsatellites is chromosome arm:nucleotide coordinate. Microsatellite data were used for estimates of pairwise Fst among colonies and the wild source population. Pairwise Fst values were calculated using Genepop [33] and neighbour joining trees were constructed using Mega 2.1 [34]. Estimates of founder colony diversity were performed on mosquito samples 3–5 months prior to mapping by pooled sequencing, thus diversity and divergence as shown in Fig. 1 should accurately represent diversity present at the mapping stage.

### *P. falciparum* gametocyte culture and mosquito infection

*P. falciparum* isolate NF54 was cultured using an automated tipper-table system [35] as implemented in the CEPIA mosquito infection facility of the Institut Pasteur [24]. For infection, mature gametocytes are added to fresh erythrocytes in AB human serum, mixed gently, and transferred to a membrane feeder prewarmed to 37 °C. Mosquitoes were allowed to feed for 15 min, and only fully engorged females were used for further analysis.

### Measurement of mosquito infection phenotypes

Infection phenotypes were oocyst infection prevalence and intensity. Oocyst prevalence is the fraction of mosquitoes carrying at least one oocyst, while intensity is the number of oocysts per mosquito determined only in the subset of mosquitoes with ≥1 oocyst. Midguts of bloodfed females were dissected 7–8 days post-infection, stained in 1xPBS buffer with 0.4 % mercury dibromofluorescein (Sigma) and the number of oocysts per midgut was determined by light microscopy. Carcasses of the dissected mosquitoes were stored at −20 °C until DNA extraction. Genomic DNA was extracted from individual female mosquitoes by homogenization in 100ul DNAZol (Invitrogen, CA, USA) using a disposable pestle, following the manufacturer’s protocol.

### Genome sequencing of mosquito phenotype pools

Based on the observed number of a *P. falciparum* oocysts, mosquitoes were assigned to one of three phenotype categories, and phenotype pools were constituted from ≥14 mosquitoes each for i) the “Zero” pool of bloodfed mosquitoes carrying zero oocysts, ii) the “Low” pool with 1–6 oocysts, and iii) the “High” pool with ≥10 oocysts. Thresholds for phenotype pools are determined empirically. Specifically for Fd03, the zero pool was comprised of 20 mosquitoes, the low pool (carrying 1–5 oocysts) included 17 mosquitoes and the high pool (>10 oocysts) included 14 mosquitoes. The entire infection comprised 93 individuals and the pools included 51, or 55 %. For Fd09, each pool was comprised of 20 individuals, with the low pool defined as 1–6 oocysts and the high pool as >29 oocysts. The complete infection had 102 individuals and thus the pools comprised 59 % of the entire infection. DNA concentrations were determined by the picogreen method [36], and DNAs of individual mosquitoes were combined at equal molarity to obtain a total of 700 ng per phenotype pool. The pooled DNAs were submitted to Illumina sequencing and sequenced to an average depth of 40× per pool or ~ 2× per mosquito.

### Genome-wide mapping by loss-of-diversity measurement

Illumina sequences were aligned to the AgamP3 genome [20] using Bowtie version 0.12.7 [37]. Reads with low mapping quality (MQ < 40) were removed and allele frequencies called using samtools mpileup [38]. Apparent low frequency variants, which could be either true low frequency alleles or sequencing errors, are irrelevant in a windowed analysis of pooled samples, and were not resolved. Pooled heterozygosity was calculated across sliding windows (10 kb windows, 1 kb steps) for each of the phenotype pools individually, as well as for the whole founder colony combined, using the *Hp* metric proposed by Qanbari *et al*. [39]. Relative diversity (*HpR*) was calculated as the proportion of heterozygosity in a phenotype pool relative to total heterozygosity within the whole founder colony after normalising for overall read-depth in each pool. Standard deviation of *HpR* values (*SHpR*) was used to identify regions with over-represented haplotypes as compared to the whole founder colony. High-*SHpR* regions within ≤5 Mb were combined to constitute a single locus.

To establish significance thresholds, random resampling was performed for 1000 permutations for each window. S*HpR* values were then segmented using the *fastseg* Bioconductor [40] package to identify clearly differing regions. Regions below 1e^−4^ probability according to the permutation analysis were removed. Three regions were selected for subsequent fine mapping: two from Fd09 and one from Fd03.

$$ \begin{array}{c}\hfill \begin{array}{c}\hfill Ni = \mathrm{read}\ \mathrm{depth}\ \mathrm{at}\ \mathrm{locus}i\hfill \\ {}\hfill ni = \mathrm{major}\ \mathrm{allele}\ \mathrm{depth}\ \mathrm{at}\ \mathrm{locus}i\hfill \end{array}\hfill \\ {}\hfill Hp=\frac{2{\displaystyle {\sum}_{i=1}^1}n\mathrm{i}\kern0.5em {\displaystyle {\sum}_{i=1}^1}\left(Ni-ni\right)}{{\left({\displaystyle {\sum}_{i=1}^1}n\mathrm{i} + {\displaystyle {\sum}_{i=1}^1}\left(Ni-ni\right)\right)}^2}\hfill \\ {}\hfill \begin{array}{l}HpP = \mathrm{pool}Hp\\ {}HpT = \mathrm{total}Hp\end{array}\hfill \\ {}\hfill HpR = {\displaystyle \sum_{i=1}^l}\frac{\left( HpPi - \overline{HpP}\right)}{HpTi}\hfill \end{array} $$

### Fine mapping by Sequenom genotyping

Loci identified from pooled sequence during the genome-wide mapping phase were filtered on the basis of differences in the proportion of reads showing the alternate allele (used here as a proxy for minor allele frequency). SNPs with the greatest differences in read-counts between phenotype pools were used to design SNP plexes for genotyping using the Sequenom MassARRAY platform. A single plex (20–25 individual SNP assays) was designed for each locus. Individual DNAs from the same experimental infection that was pool-sequenced, including individuals used to generate the pools and additional samples that did not contribute to the phenotype pools, were typed with SNPs specific to that founder colony. For both Fd03 and Fd09 there were 42 individuals that were SNP genotyped, but had not been part of the original extreme pools. These individuals either had zero oocysts or phenotypes intermediate between the low and high pools. In addition, a second, completely independent experimental infection of the same founder colony that had not been subjected to pooled sequencing was genotyped in the same way. This independent infection of Fd03 had 41 individual mosquitoes whose infection levels varied from 0 to 23 oocysts. Correlation between allele frequencies derived from pooled sequencing and individual genotyping via Sequenom is presented in Additional file 6.

Individual mosquitos were categorized into binary phenotypes with respect to infection prevalence (uninfected/infected) and infection intensity (low infected/high infected) using the same oocyst cutoffs employed for pooling. Logistic regression was used to test for significant association with phenotype using PLINK [41] and all statistics controlled for multiple testing. Replicate infections were tested for significance both individually and across replicates. Pool sequencing is a relatively young variant of whole genome sequencing, and it is a challenge to ascertain candidate SNP assays for individual genotyping, which may limit the efficiency of replicating pooled sequence loci using individual genotyping [42].

### Locus characterisation and 2Rb inversion typing

Putative variants were filtered for sequencing quality, and consequences of variants were called for both colonies using the Ensembl Variant Effect Predictor (v2.3) [43] against VectorBase genebuild AgamP3.5 [44] and using Ensembl API 65.3 (Dec 2011). Enrichment for gene ontology terms was calculated by Fisher’s exact test using custom R scripts and topGO, from the Bioconductor suite [45]. *d*N:*d*S ratios were assessed by locus counting using custom R scripts. Due to the lack of available codon substitution data for this species, multiple substitutions or codon bias could not be analysed in *d*N:*d*S results. Molecular karyotyping of the 2Rb inversion for Fd09 was carried out by a published method [46]*.* Molecular karyotyping results were confirmed against a panel of individuals previously karyotyped by polytene chromosome analysis (not shown).

### Gene functional assays and statistical analysis

Double-stranded RNAs were synthesized from PCR amplicons using the T7 Megascript Kit (Life Technologies) as described previously [24]. Primers are listed in Additional file 7. For each targeted gene, 500 ng of dsRNA (but not more than 207 nl volume) were injected into the thorax of cold-anesthetized 1-day-old *A. gambiae* females using a nanoinjector (Nanoject II; Drummond). The efficiency of gene silencing was monitored 4 d after dsRNA injection as follows. cDNA synthesis was performed using the M-MLV reverse transcriptase with random hexamers (Invitrogen). In each case, 1 μg of total RNA was used in triplicate reactions. The triplicates were pooled and the mixture was used as template for PCR analysis. Primers used in PCR for gene silencing verification are listed in Additional file 7. Verification of gene silencing is shown in Additional file 8.

Midgut oocysts were counted as described above, and were analysed for the same two phenotypes, infection prevalence and oocyst intensity. Oocyst infection values for gene silencing experiments were calculated from replicates of ≥30 dissected mosquitoes. All replicates per condition were analysed for oocyst infection prevalence. In contrast, for analysis of oocyst intensity, only the mosquitoes carrying ≥1 oocyst are considered. Therefore, for analysis of differences in oocyst intensity, a threshold of ≥30 % oocyst infection prevalence per replicate was imposed [2, 32]. For statistical analysis, comparisons of infection prevalence were made using the Chi Square test, and comparisons of oocyst intensity (excluding mosquitoes with zero oocysts) using the non-parametric Wilcoxon Mann Whitney test. At least two independent replicate infections were performed per condition. Replicates were analysed independently using the tests described above. If at least one replicate met the significance criterion of *p* ≤ 0.05, a third replicate was done. The p-values from independent tests of significance were combined using the meta-analytical approach of Fisher [47], and this combined p value is reported here. The threshold for combined significance was defined as *p* = 0.01.

## Availability of supporting data

All supporting data are included as additional files.

## Additional files

Additional file 1: Figure S1. Genome wide total heterozygosity and relative heterozygosity for founder colony Fd03. Plots depict heterozygosity measures for colony Fd03 across all chromosomes. Total pooled heterozygosity (*Hp*) was calculated in a sliding 10 kb window along the chromosome within the Fd03 colony. Dots indicate minimum and maximum values for a 1 Mb window, the black line indicates the average heterozygosity and the gray shading represents the standard deviation of total pooled heterozygosity across a 1 Mb window. Relative diversity (*HpR*) per 1 Mb window, calculated as the proportion of heterozygosity in a given pool relative to total heterozygosity within the source Fd03 colony. Color of point indicates per window elevated heterozygosity (blue), or reduced heterozygosity (red), plotted as log base 10 of the relative diversity. Phenotype pool identity is indicated in the lower left of each panel (high, low, zero oocysts). A relative heterozygosity value of 1 indicates the same heterozygosity levels in tested pool as compared to all other pools, values greater than 1 indicate greater heterozygosity in the tested pool and values less than 1 indicate lower heterozygosity in the tested pool. Given the log scale values of 0.5 and 2.0 are equidistant from 1. Candidate locus 3.1 is indicated by the red vertical shaded bar at coordinates 17.4-19.1 Mb. In this interval, relative heterozygosity is increased in the high pool and simultaneously reduced in the low and zero pools. Pools were comprised of DNA from 20 (zero oocyst pool), 17 (low oocyst pool), or 14 (high oocyst pool) individuals. (PDF 4909 kb) Additional file 2: Figure S2. Genome wide total heterozygosity and relative heterozygosity for founder colony Fd09. Plots depict heterozygosity measures for colony Fd09 across all chromosomes. Total pooled heterozygosity (*Hp*) was calculated in a sliding 10 kb window along the chromosome within the Fd09 colony. Dots indicate minimum and maximum values for a 1 Mb window, the black line indicates the average heterozygosity and the gray shading represents the standard deviation of total pooled heterozygosity across a 1 Mb window. Relative diversity (*HpR*) per 1 Mb window, calculated as the proportion of heterozygosity in a given pool relative to total heterozygosity within the source Fd09 colony. Color of point indicates per window elevated heterozygosity (blue), or reduced heterozygosity (red), plotted as log base 10 of the relative diversity. Phenotype pool identity is indicated in the lower left of each panel (high, low, zero oocysts). A relative heterozygosity value of 1 indicates the same heterozygosity levels in tested pool as compared to all other pools, values greater than 1 indicate greater heterozygosity in the tested pool and values less than 1 indicate lower heterozygosity in the tested pool. Given the log scale values of 0.5 and 2.0 are equidistant from 1. Candidate loci 9.1 and 9.2 are indicated by dark blue and light blue shading, respectively, on Chromosome 2. At locus 9.1, relative heterozygosity is decreased in the high pool and simultaneously reduced in the low and zero pools, while at locus 9.2, relative heterozygosity in increased in the high pool and decreased in the other pools. Each pool was comprised of DNA from 20 individual mosquitoes. (PDF 4973 kb) Additional file 3: Table S1. Fd03 Infection Prevalence Association and Table S2. Fd03 Infection Intensity Association (XLSX 15 kb) Additional file 4: Figure S3. Manhattan plot for colony Fd03. Results are given for the two replicates individually and the values combined by Fisher’s method. All genotyped loci were tested for association with two phenotypes by logistic regression using PLINK. Infection prevalence (blue) was defined as having more than one oocyst in the dissected midgut, infection intensity (red, measured only for mosquitoes carrying ≥1 oocyst) as having >5 oocysts. Dashed line represents a 0.01 p-value (i.e., 1/p = 100). Genes in the regions are shown below; *Toll11* and *Toll10* are the two rightmost genes on the positive strand. The combined plot represents values from reps 1 and 2 combined by the method of Fisher [47]. (PDF 127 kb) Additional file 5: Table S3. All predicted genes within the mapped locus, Pfin 7. (XLSX 13 kb) Additional file 6: Figure S4. Concordance of allele frequencies as called from pooled sequence and Sequenom individual genotyping. Concordance was assessed with data from three Sequenom plexes spread across all 3 candidate loci, 3.1, 9.1, and 9.2. SNPs were chosen independently of the phenotype group; that is, they were chosen on the basis of differing allele frequencies in either of the founder pools, and not for high allele differences between phenotype pools. Allele frequencies for Sequenom data were calculated from genotype calls and allele frequencies from pool sequence were determined by relative read depth. Dot color indicates the founder colony and phenotype group as indicated in the legend. (PDF 113 kb) Additional file 7: Table S4. Primers for gene silencing and efficiency verification. (XLSX 10 kb) Additional file 8: Figure S5. Verification of gene silencing for Toll10 and Toll 11. Four days after dsRNA injection (indicated above gel), cDNA synthesis was performed using M-MLV reverse transcriptase with random hexamers (Invitrogen). In each case, 1ug of total RNA was used in triplicate assays. Triplicates were pooled and used a template for PCR analysis of targeted (TOLL 10, TOLL 11) and control (ribosomal protein Rps7) genes (indicated on left margin of gel). (PDF 192 kb)
