# Supplementary figures and images for: Association mapping by pooled sequencing identifies TOLL 11 as a protective factor against Plasmodium falciparum in Anopheles gambiae
Source: BMC Genomics. 2015 Oct 13;16:779. doi: 10.1186/s12864-015-2009-z (PMC4603968; doi:10.1186/s12864-015-2009-z)

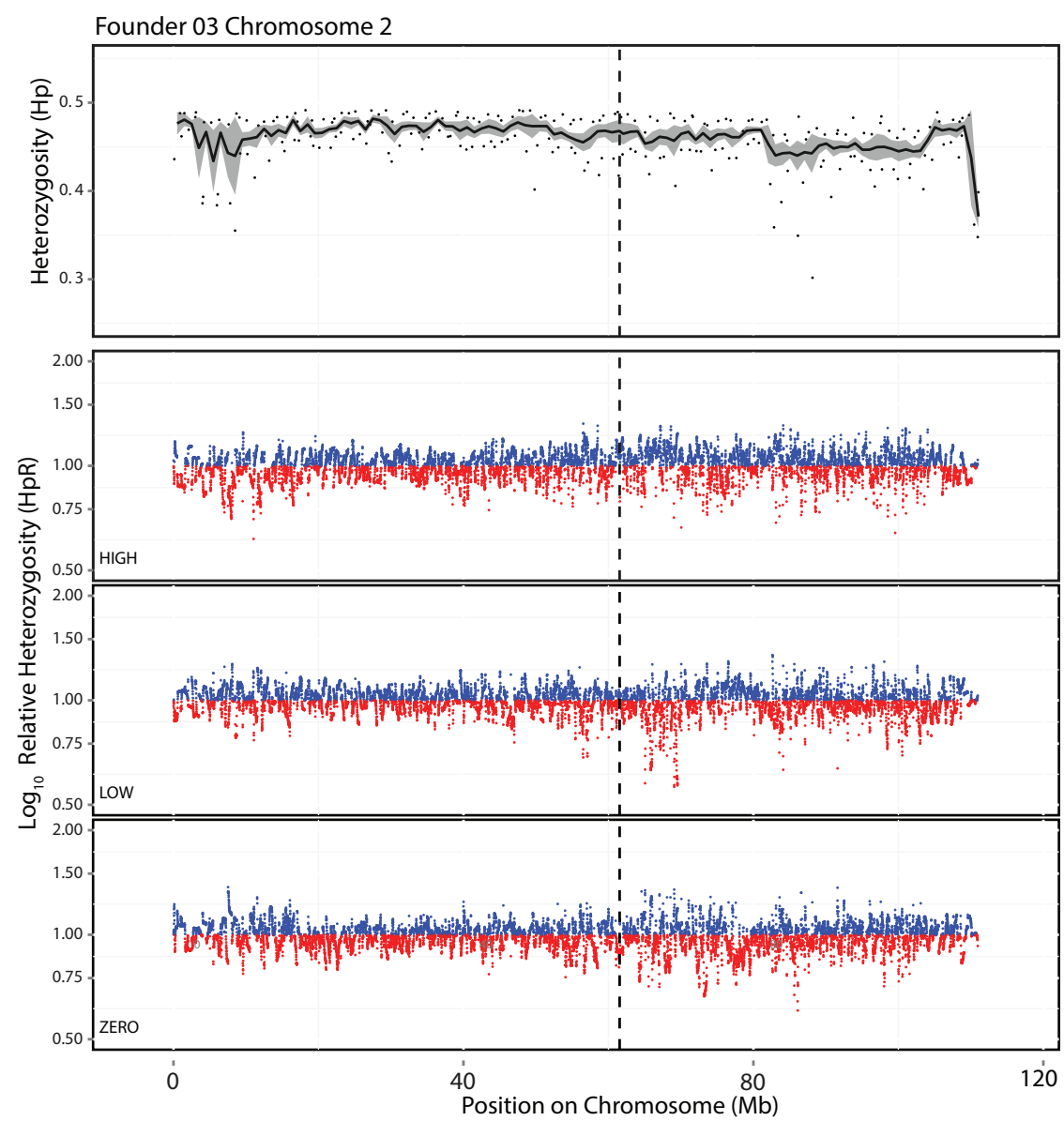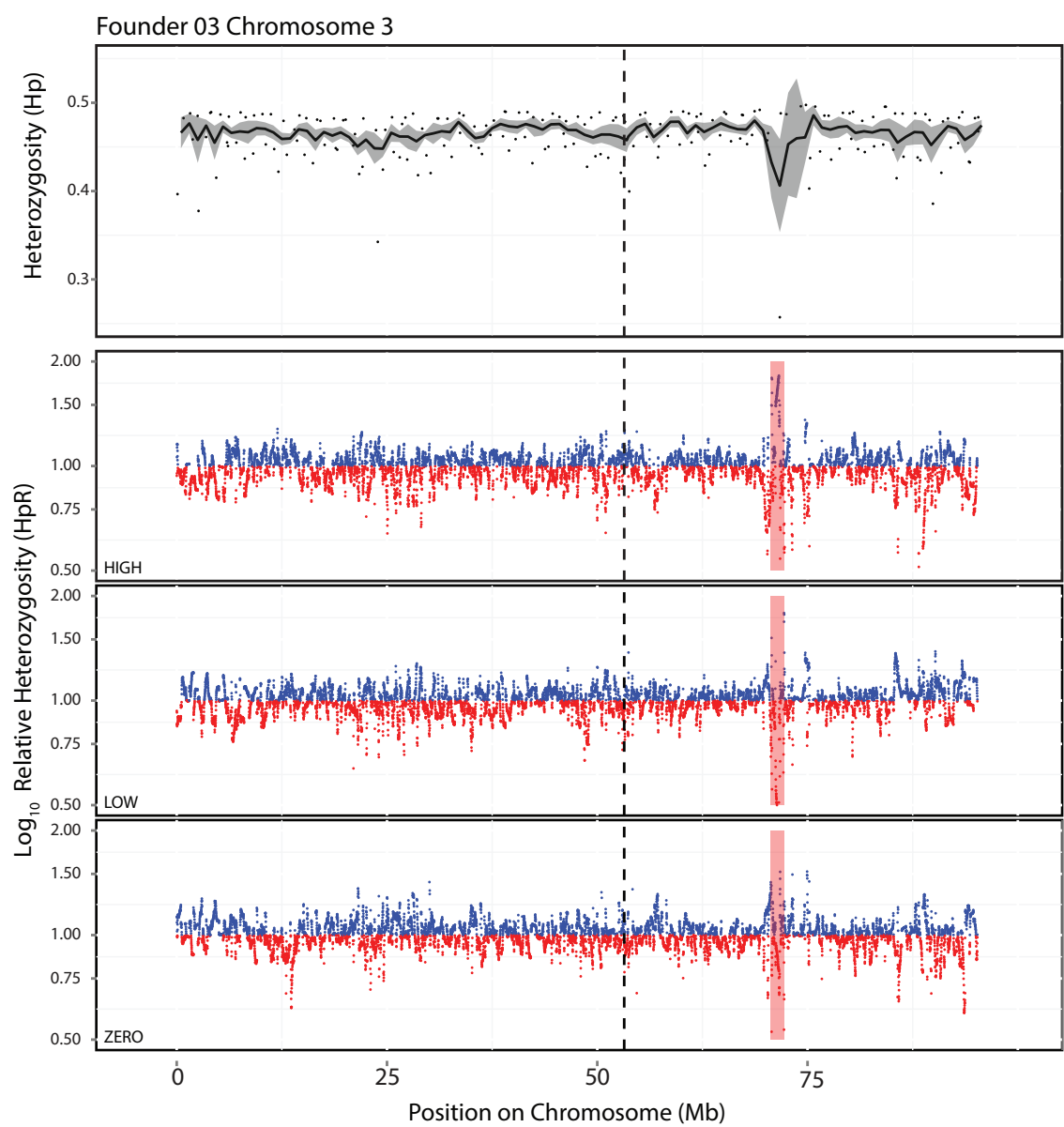

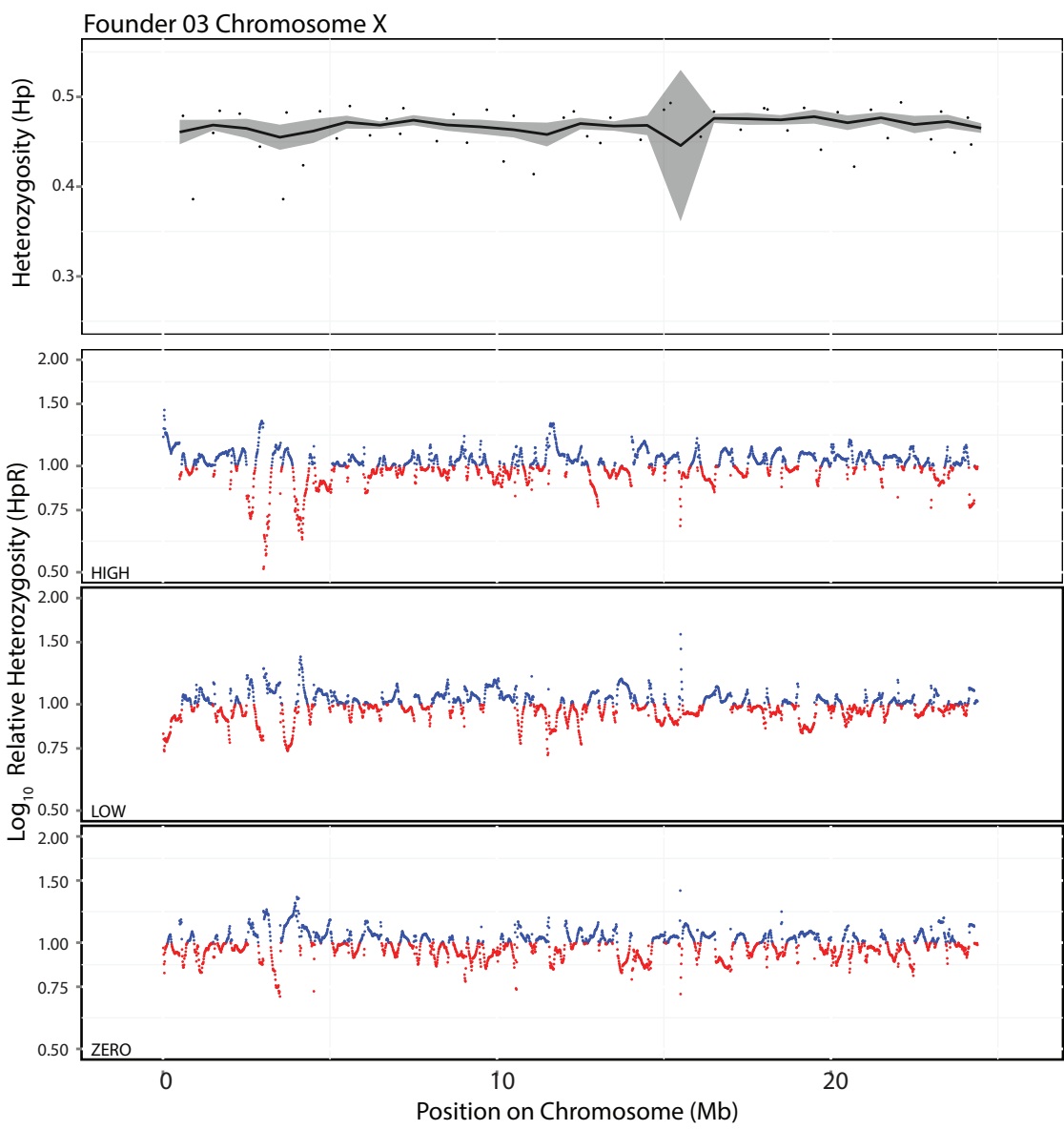

Supplement: Additional file 1: Figure S1. — Genome wide total heterozygosity and relative heterozygosity for founder colony Fd03. Plots depict heterozygosity measures for colony Fd03 across all chromosomes. Total pooled heterozygosity (Hp) was calculated in a sliding 10 kb window along the chromosome within the Fd03 colony. Dots indicate minimum and maximum values for a 1 Mb window, the black line indicates the average heterozygosity and the gray shading represents the standard deviation of total pooled heterozygosity across a 1 Mb window. Relative diversity (HpR) per 1 Mb window, calculated as the proportion of heterozygosity in a given pool relative to total heterozygosity within the source Fd03 colony. Color of point indicates per window elevated heterozygosity (blue), or reduced heterozygosity (red), plotted as log base 10 of the relative diversity. Phenotype pool identity is indicated in the lower left of each panel (high, low, zero oocysts). A relative heterozygosity value of 1 indicates the same heterozygosity levels in tested pool as compared to all other pools, values greater than 1 indicate greater heterozygosity in the tested pool and values less than 1 indicate lower heterozygosity in the tested pool. Given the log scale values of 0.5 and 2.0 are equidistant from 1. Candidate locus 3.1 is indicated by the red vertical shaded bar at coordinates 17.4-19.1 Mb. In this interval, relative heterozygosity is increased in the high pool and simultaneously reduced in the low and zero pools. Pools were comprised of DNA from 20 (zero oocyst pool), 17 (low oocyst pool), or 14 (high oocyst pool) individuals. (PDF 4909 kb) [file 12864_2015_2009_MOESM1_ESM.pdf]

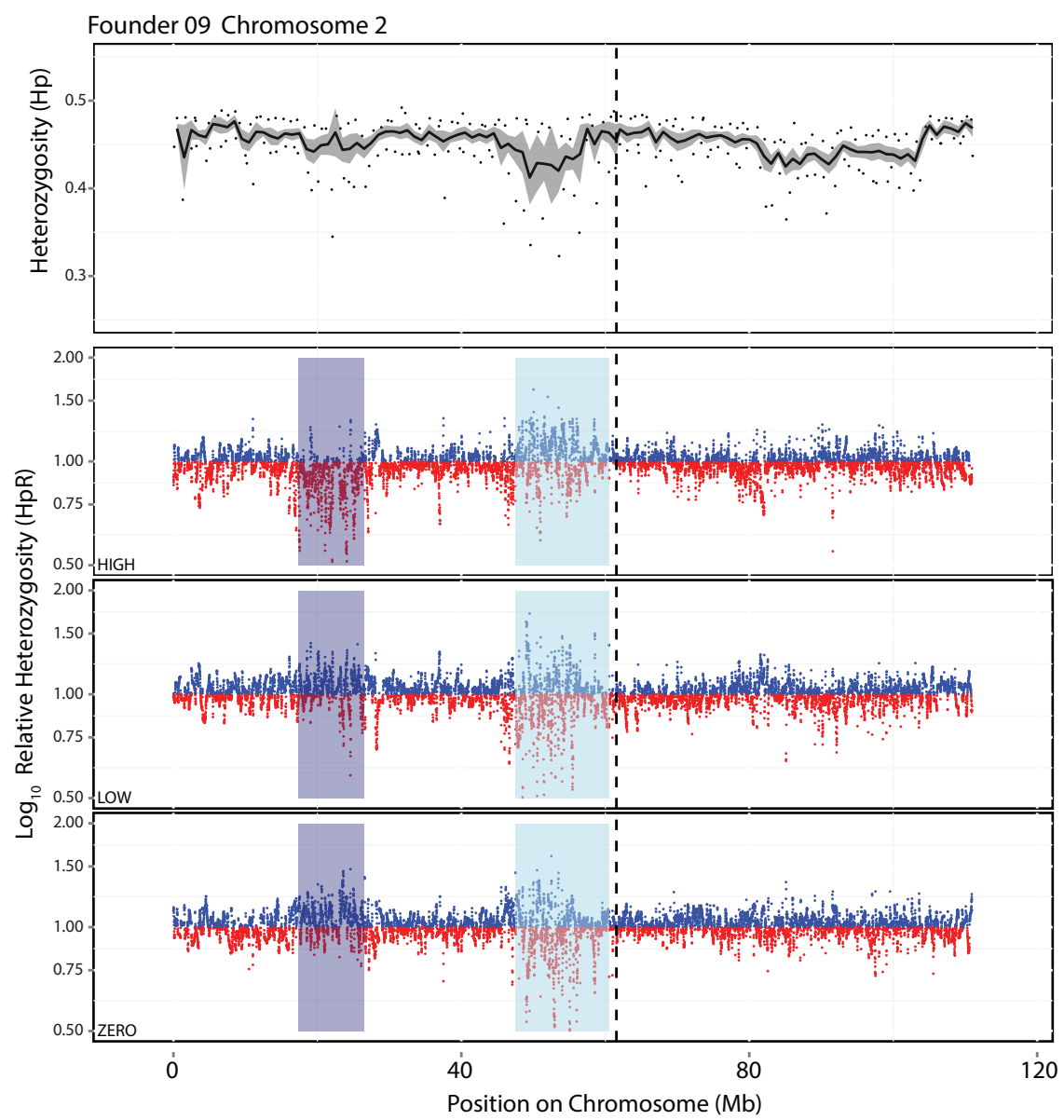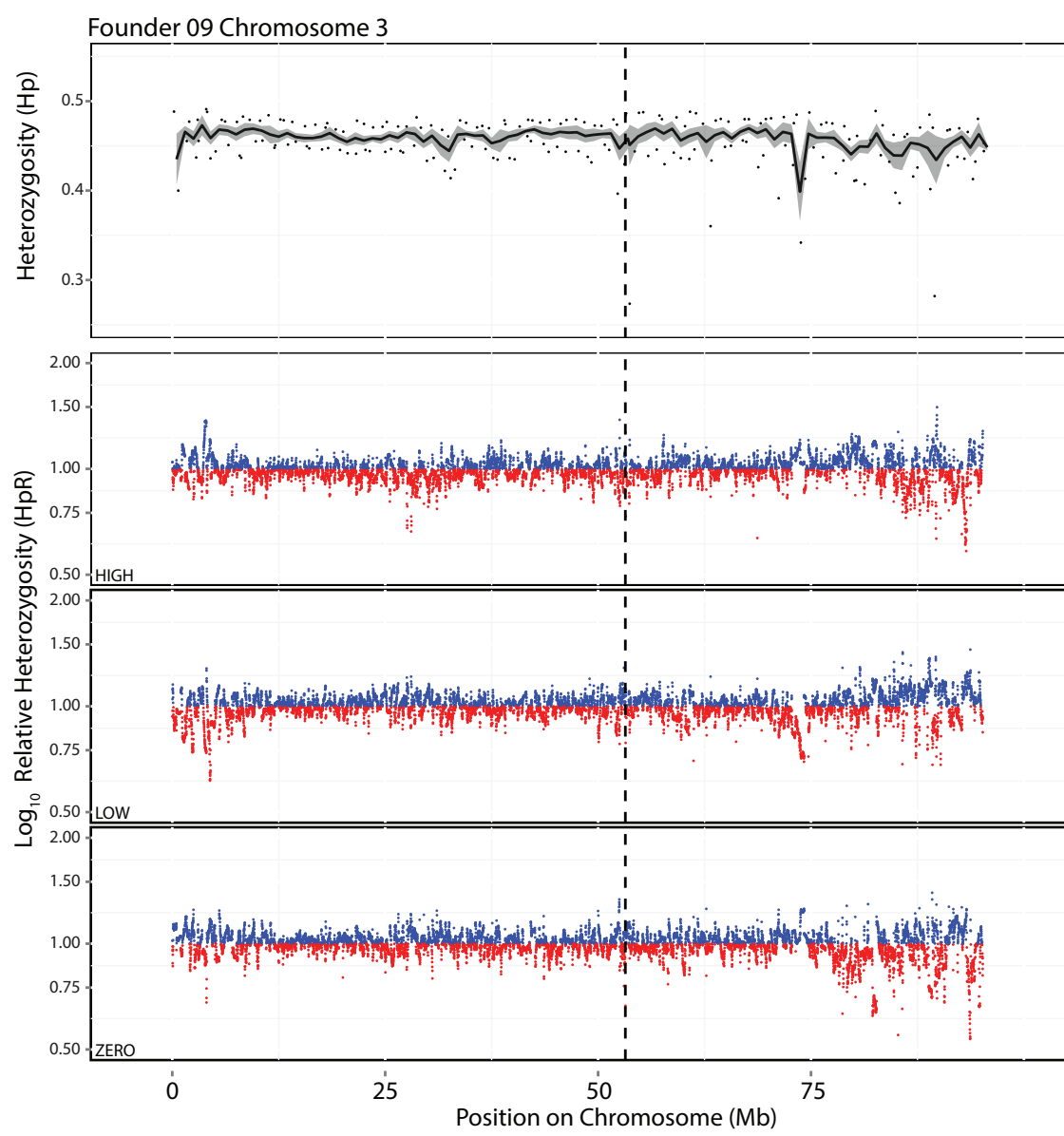

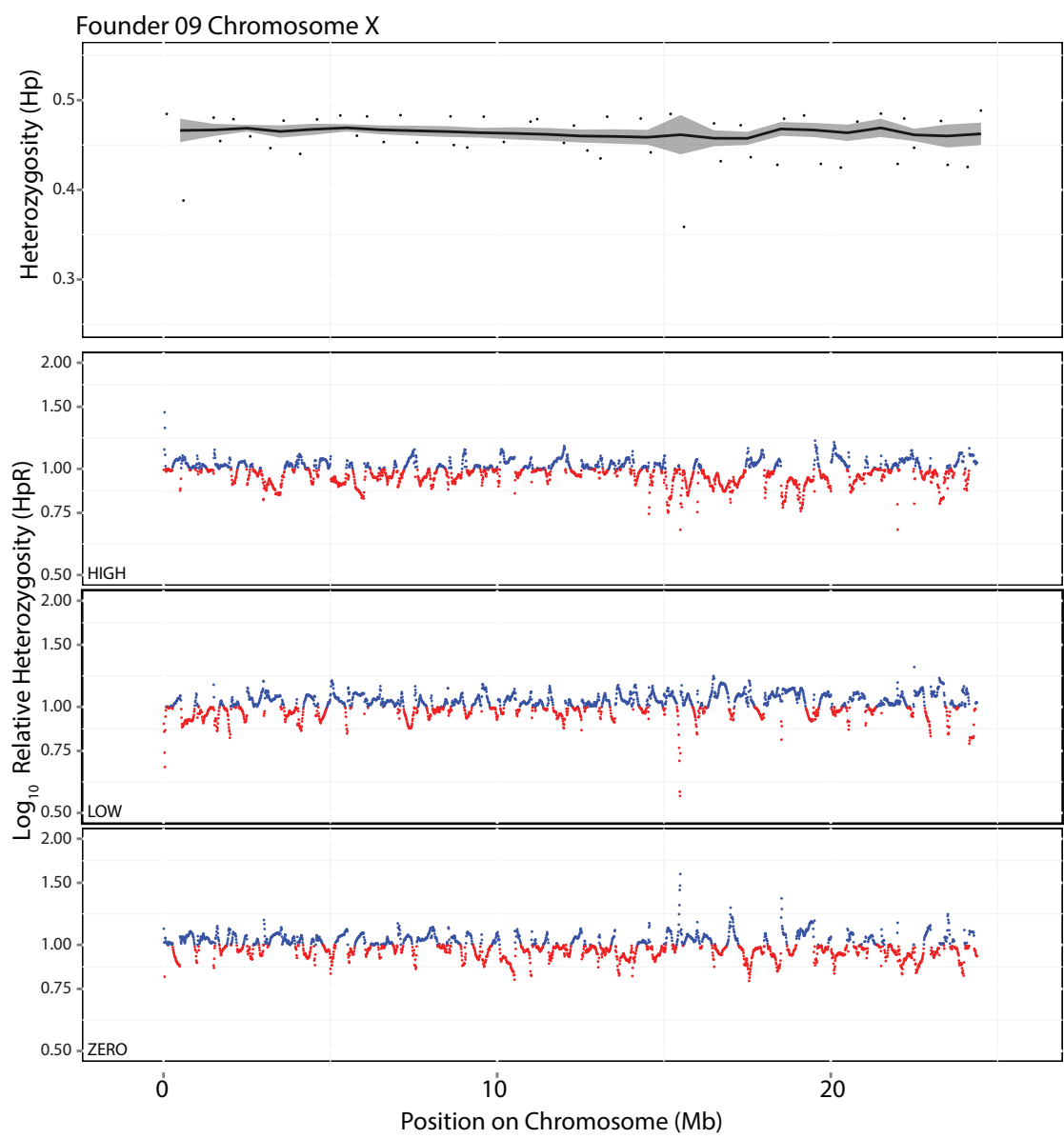

Supplement: Additional file 2: Figure S2. — Genome wide total heterozygosity and relative heterozygosity for founder colony Fd09. Plots depict heterozygosity measures for colony Fd09 across all chromosomes. Total pooled heterozygosity (Hp) was calculated in a sliding 10 kb window along the chromosome within the Fd09 colony. Dots indicate minimum and maximum values for a 1 Mb window, the black line indicates the average heterozygosity and the gray shading represents the standard deviation of total pooled heterozygosity across a 1 Mb window. Relative diversity (HpR) per 1 Mb window, calculated as the proportion of heterozygosity in a given pool relative to total heterozygosity within the source Fd09 colony. Color of point indicates per window elevated heterozygosity (blue), or reduced heterozygosity (red), plotted as log base 10 of the relative diversity. Phenotype pool identity is indicated in the lower left of each panel (high, low, zero oocysts). A relative heterozygosity value of 1 indicates the same heterozygosity levels in tested pool as compared to all other pools, values greater than 1 indicate greater heterozygosity in the tested pool and values less than 1 indicate lower heterozygosity in the tested pool. Given the log scale values of 0.5 and 2.0 are equidistant from 1. Candidate loci 9.1 and 9.2 are indicated by dark blue and light blue shading, respectively, on Chromosome 2. At locus 9.1, relative heterozygosity is decreased in the high pool and simultaneously reduced in the low and zero pools, while at locus 9.2, relative heterozygosity in increased in the high pool and decreased in the other pools. Each pool was comprised of DNA from 20 individual mosquitoes. (PDF 4973 kb) [file 12864_2015_2009_MOESM2_ESM.pdf]

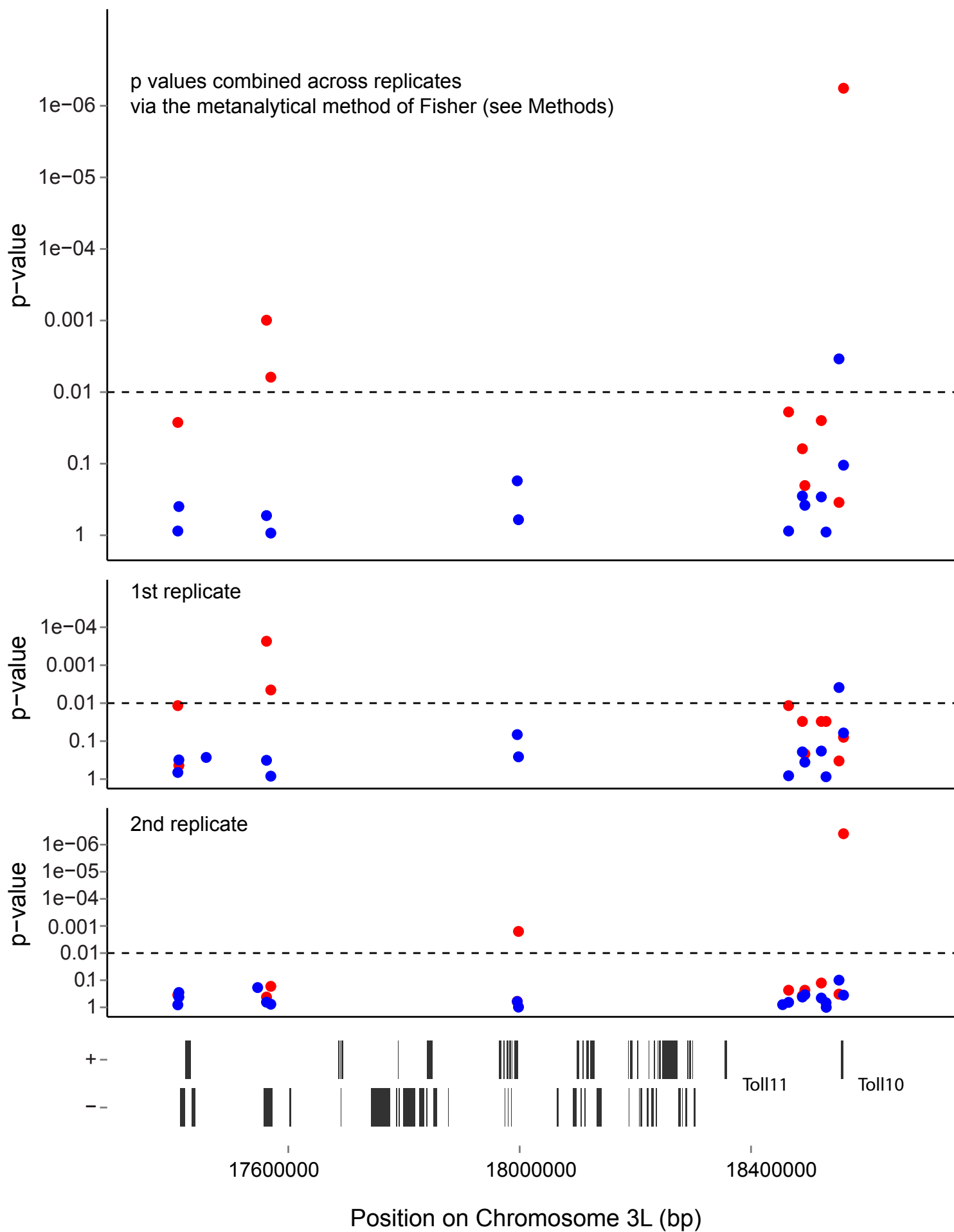

Supplement: Additional file 4: Figure S3. — Manhattan plot for colony Fd03. Results are given for the two replicates individually and the values combined by Fisher’s method. All genotyped loci were tested for association with two phenotypes by logistic regression using PLINK. Infection prevalence (blue) was defined as having more than one oocyst in the dissected midgut, infection intensity (red, measured only for mosquitoes carrying ≥1 oocyst) as having >5 oocysts. Dashed line represents a 0.01 p-value (i.e., 1/p = 100). Genes in the regions are shown below; Toll11 and Toll10 are the two rightmost genes on the positive strand. The combined plot represents values from reps 1 and 2 combined by the method of Fisher [47]. (PDF 127 kb) [file 12864_2015_2009_MOESM4_ESM.pdf]

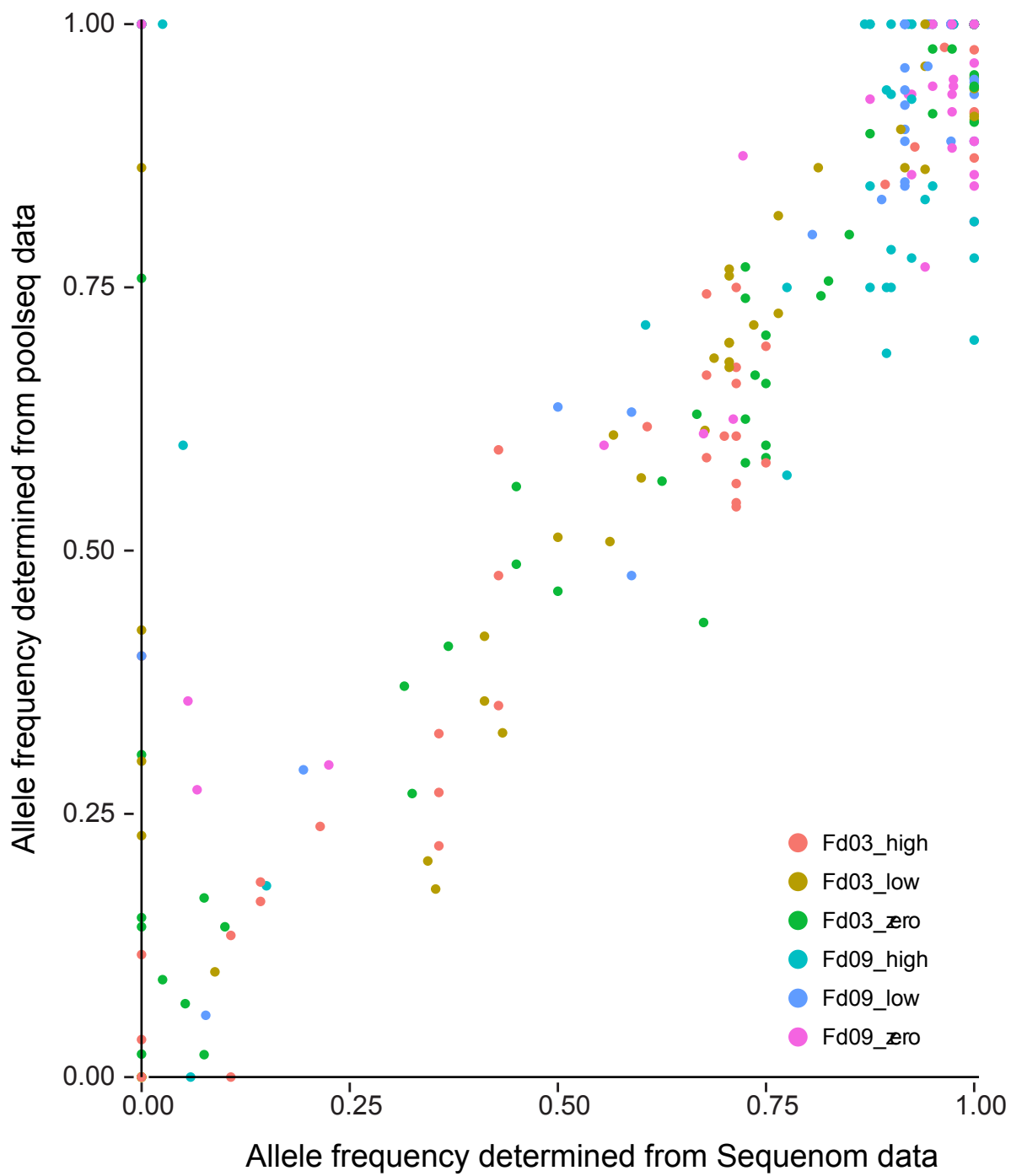

Supplement: Additional file 6: Figure S4. — Concordance of allele frequencies as called from pooled sequence and Sequenom individual genotyping. Concordance was assessed with data from three Sequenom plexes spread across all 3 candidate loci, 3.1, 9.1, and 9.2. SNPs were chosen independently of the phenotype group; that is, they were chosen on the basis of differing allele frequencies in either of the founder pools, and not for high allele differences between phenotype pools. Allele frequencies for Sequenom data were calculated from genotype calls and allele frequencies from pool sequence were determined by relative read depth. Dot color indicates the founder colony and phenotype group as indicated in the legend. (PDF 113 kb) [file 12864_2015_2009_MOESM6_ESM.pdf]

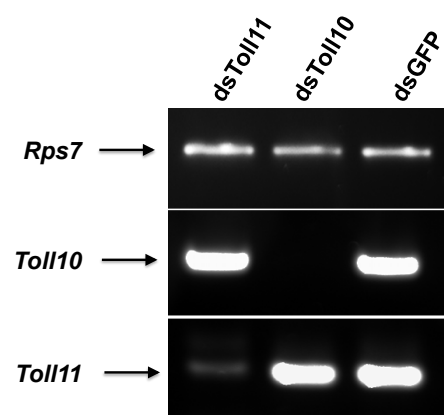

Supplement: Additional file 8: Figure S5. — Verification of gene silencing for Toll10 and Toll 11. Four days after dsRNA injection (indicated above gel), cDNA synthesis was performed using M-MLV reverse transcriptase with random hexamers (Invitrogen). In each case, 1ug of total RNA was used in triplicate assays. Triplicates were pooled and used a template for PCR analysis of targeted (TOLL 10, TOLL 11) and control (ribosomal protein Rps7) genes (indicated on left margin of gel). (PDF 192 kb) [file 12864_2015_2009_MOESM8_ESM.pdf]
